# Antiphospholipid Antibodies Associated with Native Arteriovenous Fistula Complications in Hemodialysis Patients: A Comprehensive Review of the Literature

**DOI:** 10.3390/antib13010001

**Published:** 2024-01-02

**Authors:** Maxime Taghavi, Abla Jabrane, Lucas Jacobs, Maria Do Carmo Filomena Mesquita, Anne Demulder, Joëlle Nortier

**Affiliations:** 1 Nephrology and Dialysis Department, Brugmann University Hospital, Université Libre de Bruxelles (ULB), 1020 Brussels, Belgium; abla.jabrane@ulb.be (A.J.); lucas.jacobs@chu-brugmann.be (L.J.); maria.mesquita@chu-brugmann.be (M.D.C.F.M.); joelle.nortier@chu-brugmann.be (J.N.); 2 Laboratory of Experimental Nephrology, Faculty of Medicine, Université Libre de Bruxelles (ULB), 1070 Brussels, Belgium; 3 Laboratory of Hematology and Haemostasis LHUB-ULB, Brugmann University Hospital, Université Libre de Bruxelles (ULB), 1020 Brussels, Belgium; anne.demulder@chu-brugmann.be

**Keywords:** antiphospholipid antibodies, antiphospholipid syndrome, arteriovenous fistula, hemodialysis, thrombosis, stenosis, maturation failure

## Abstract

Antiphospholipid antibody (aPL)-persistent positivity is frequent in hemodialysis (HD) patients. Native arteriovenous fistula (AVF) complications such as stenosis and thrombosis are among the most important causes of morbidity and mortality in hemodialysis patients. The association between aPL positivity and AVF thrombosis seems to now be well established. However, whether aPL positivity is associated with other AVF complications, such as maturation failure or stenosis, is not well known. Given the significant impact of AVF failure on patient’s prognosis, it is of interest to further investigate this particular point in order to improve prevention, surveillance and treatment, and, ultimately, the patient’s outcome. This literature review aims to report the recent literature on aPL-associated native AVF complications.

## 1. Introduction

### 1.1. Hemodialysis and Vascular Access

End-stage kidney disease (ESKD) is typically defined by a glomerular filtration rate of less than 15 mL/min/1.73 m^2^ and requires the initiation of renal replacement therapy, such as hemodialysis (HD), peritoneal dialysis, or kidney transplantation, for survival [1]. The incidence of ESKD is increasing worldwide and the large majority of the patients remain on chronic HD, a modality requiring an efficient vascular access. The options for vascular access in HD patients include native arteriovenous fistulas (AVF), arteriovenous grafts (AVG), and tunneled central venous catheters (CVC). Native AVF is generally considered as the best option for vascular access in HD patients, because of lower rates of infection and thrombosis compared to AVG and CVC. Additionally, AVF have been associated with improved long-term survival and reduced healthcare costs [2,3]. However, AVF complications are common in HD patients and can lead to significant morbidity and mortality. These complications include thrombosis, stenosis, infection, aneurysm, pseudoaneurysm, and hemorrhage. Thrombosis and stenosis are the most common complications, often requiring intervention with angioplasty or thrombectomy. Regular monitoring and timely intervention can help prevent and manage these complications [4]. 

### 1.2. Antiphospholipid Syndrome and Pathophysiology

Antiphospholipid syndrome (APS) is an autoimmune disorder characterized by the persistent positivity of circulating antiphospholipid antibodies (aPL) resulting in arterial, venous, or microvascular thrombosis and obstetrical complications. The pathophysiology of thrombosis in APS is complex and multifactorial. aPL trigger phospholipids and phospholipid binding proteins at different cell surfaces (i.e., endothelial cell, platelets, monocytes, and neutrophils). Endothelial cells are activated by aPL and acquire a phenotype that promotes inflammation, complement activation, leukocyte trafficking, and a procoagulant state. This activation ultimately leads to in situ thrombosis while also promoting other non-thrombotic autoimmune and inflammatory complications [5,6]. APS have also been associated with endothelial cell dysfunction both in vitro and in vivo [7,8]. Distinct from thrombotic events, the chronic occlusive APS vasculopathy is characterized by cell proliferation and infiltration that progressively expands the intima, therefore narrowing the vascular lumen. The latter was first described in aPL nephropathy [9]. The mammalian Target of Rapamycin (mTOR) pathway, implicated in cell proliferation and survival, seems to be an important signaling pathway by which aPL trigger intimal hyperplasia and occlusive vasculopathy [6,10]. 

A “two-hit” model in the pathogenesis of APS has been proposed, postulating that aPL provide the first hit favoring a procoagulant state but not sufficient to cause thrombosis. Subsequently, a second hit (e.g., an infectious or inflammatory stimuli or a vascular injury) will lead to vascular thrombosis. This second hit is not obvious in many cases [6]. 

### 1.3. Classification Criteria of Antiphospholipid Syndrome

The 2006 Revised Sapporo APS classification criteria have been recently revised in 2023 by the American College of Rheumatology (ACR) and the European Alliance of Associations for Rheumatology (EULAR) [11,12]. These new classification criteria are based on a scoring system for both laboratory and clinical criteria. Patients can be classified as APS for research purposes if there are at least 3 points from clinical domains and at least 3 points from laboratory domains. As in the previous criteria, aPL positivity must be confirmed after at least 12 weeks. Three aPL assays are recommended, including Immunoglobulin (Ig) G or IgM anticardiolipin antibody (aCL), IgG or IgM anti-beta2 glycoprotein I antibody (aβ2-GPI), or Lupus Anticoagulant (LA). The 2023 ACR/EULAR classification criteria no longer consider isolated positivity of IgM aCL or IgM aβ2-GPI as sufficient [11,12]. Other non-criteria antibodies potentially predictive of thrombosis in APS such as IgA aCL; IgA aβ2-GPI; IgG, IgA, IgM anti-phosphatidylserine/prothrombin (aPS/PT); IgG anti-phosphatidylserine antibodies (aPS) are not included as well [12,13]. 

With respect to the clinical manifestations, the new 2023 ACR/EULAR APS classification criteria allow for the stratification of risk for macrovascular events through the assessment of traditional thrombosis risk factors with weighted assessment. The definitions of high-risk venous thromboembolism and cardiovascular disease are presented in the article. These criteria also define microvascular domain items considered mechanistically distinct from moderate-to-large vessel disease. Indeed, features, such as APS Nephropathy, cardiac valve disease, livedo racemose, and thrombocytopenia, have been added to better capture and quantify the diverse manifestations of APS. These new 2023 ACR/EULAR APS classification criteria have a specificity of 99% compared to the 86–91% specificity of the 2006 Revised Sapporo criteria [12]. Table 1 summarizes the main differences between 2006 Revised Sapporo criteria and 2023 ACR/EULAR classification criteria.

### 1.4. Antiphospholipid Antibodies in Hemodialysis Patients 

ESKD is rare in APS [14]. On the other hand, aPL-persistent positivity is frequently seen in ESKD. Its prevalence is higher in HD patients when compared to ESKD conservatively treated, to peritoneal dialysis patients and to general population [15]. Indeed, the prevalence of aPL in HD patients varies from 11 to 56% [16,17,18,19,20,21,22,23] and is estimated to range between 40 and 50 cases per 100,000 in the general population [24]. However, aPL positivity is inconsistently associated with AVF complications such as thrombosis and stenosis. 

In this review, we will discuss the etiopathogenic role of aPL in AVF complications in HD patients and the available treatment options. We will focus on native AVF immediate complications, maturation failure, and stenosis or thrombosis associated with aPL status. Complications related to AVG or CVC are not discussed in the present article. 

## 2. Methods 

A literature review was performed using a PubMed electronic search by using the following words: 

For AVF Maturation: arteriovenous fistula AND maturation AND (antiphospholipid antibody OR anticardiolipin antibody OR anti beta 2 glycoprotein I OR lupus anticoagulant OR antiphospholipid syndrome).

For AVF Thrombosis: arteriovenous fistula AND thrombosis AND (antiphospholipid antibody OR anticardiolipin antibody OR anti beta 2 glycoprotein I OR lupus anticoagulant OR antiphospholipid syndrome).

For AVF Stenosis: arteriovenous fistula AND stenosis AND (antiphospholipid antibody OR anticardiolipin antibody OR anti beta 2 glycoprotein I OR lupus anticoagulant OR antiphospholipid syndrome).

A total of 32 articles were found and analyzed. References of the included articles were also checked. Case reports and articles reporting only AVG or CVC were not included in the present paper. Papers in French and in English were included. 

## 3. Interpretation and Limitation of aPL Positivity in HD Patients

The possible impact of aPL on the incidence of AVF complications is the subject of contradictory findings raising the hypothesis that aPL positivity is an epiphenomenon in ESKD. Several hypotheses have been proposed. First, false-positive aPL may be observed during anticoagulation therapy, widely used in HD patients [25]. aPL positivity might also be explained by molecular mimicry as a response to the exposure to microorganisms, such as hepatitis C virus [26,27], exposure to endotoxins related either to ESKD, or to HD. Also, the role of the gut microbiota modification in ESKD has been suggested [28,29]. Hemodialysis membranes have also been associated with higher incidence of aPL (e.g., cuprophane membranes) [30]. These findings are consistent with the higher prevalence and higher titers of aPL seen in HD patients compared to conservatively treated ESKD patients [15,20,31]. The same observation is made in patients with AVG compared to native AVF [20]. Whether higher prevalence of aPL is related to the use of an AVG or if aPL positivity is associated with vessels injury and therefore the need for AVG is unknown. Some authors suggest that aPL positivity in ESKD may simply reflect a response to oxidation (i.e., cross-reactive immunoglobulins against epitopes of oxidized lipids) that worsens as ESKD progresses [15]. Interestingly, a prospective study performed by Jamshid Roozbeh et al. found that HD vintage but also a greater number of dialysis sessions were associated with a higher prevalence of aCL [17].

There are major limitations when comparing studies on aPL positivity in HD patients over the years. Indeed, during the past decades, evolution of dialysis techniques (e.g., high flux dialyzer, online hemodiafiltration, ultrapure dialysate) and improvements in membrane biocompatibility led to better patient outcomes with less inflammation, better clearance of small and middle size molecules, as well as uremic toxins [32]. Despite these changes, the prevalence of aPL positivity in HD patients did not drop [28]. 

Other limitations are related to the heterogenicity of aPL assays: (a) most of the studies focused on IgG aCL, with or without IgM aCL, (b) aβ2-GPI was proposed as an laboratory diagnosis criterion only since 2006 [11], (c) new assays for LA have been developed and new guidelines have been published [25,33], (d) different cut off are used for aCL and aβ2-GPI and some studies use a cut off below a significant clinical threshold, (e) heterogeneous units are used for aCL and aβ2-GPI, and (f) uneven use of aPL confirmation at 12 weeks. Table 2 summarizes available studies on aPL and AVF outcomes and specifies aPL assays, the cutoff used and whether aPL was confirmed.

Finally, studies on aPL profile stability over time have shown that about 10% of APS patients will experience aPL negativisation. In a prospective study on 259 APS patients, 8.9% of the patients experienced negativisation over a 5 year follow-up period. Negativisation was defined as repeated aPL measurements on at least two consecutive occasions at least 12 weeks apart, with a follow-up of at least 1 year from the time aPL first turned negative. In this study, none of the patients experienced any recurrent thrombotic event during the follow-up period after negativisation. Negativisation was mainly observed in single aPL positive patients [46,47]. Whether anticoagulation should be stopped in these patients is not known. Interestingly, in hemodialysis patients, the same observation was reported in a retrospective study on 208 patients. About 16% of the patients experienced either a negativisation or a seroconversion of aPL. However, the outcomes on vascular access thrombosis of this specific group was not reported. In this study, negativisation was defined as a negative aPL assay after only one positive assay [36]. We recently performed a retrospective study including 103 HD patients and we reported similar findings. Indeed, 20% of our cohort experienced either a negativisation or a seroconversion of aPL. Negativisation was defined as one or multiple negative aPL assays after one positive aPL assay. Interestingly, this subgroup of patients had significantly higher maturation failure rates compared to aPL negative patients. This study was designed to assess AVF thrombosis or stenosis, only during the AVF maturation period (i.e., a 6-week period) [48].

## 4. Antiphospholipid Mediated AVF Complications

### 4.1. AVF Maturation

After native AVF surgical creation, the outflow vein goes through a complex vascular remodeling process called the “maturation process”, usually taking place within 4 to 6 weeks. Actually, this time period can continue during three post-operative months [49]. The outflow vein will experience vasodilatation and wall thickening therefore allowing a two-needle puncture [50]. AVF maturation can be assessed clinically or by using ultrasound imaging mainly based on AVF blood flow and outflow vein diameter [51,52]. AVF maturation failure is a frequent complication that affects more than half of the AVF and requires frequent interventions in order to facilitate maturation (i.e., assisted maturation) [50,53]. Intimal hyperplasia is the main stenosis lesion and the leading cause of AVF non maturation [54]. It has been associated with endothelial dysfunction both in vivo and in vitro [55,56,57]. In the multicenter prospective Hemodialysis Maturation Fistula Study, interventions performed for AVF stenosis were the most frequent interventions aiming to facilitate AVF maturation [53].

**Figure 1:**
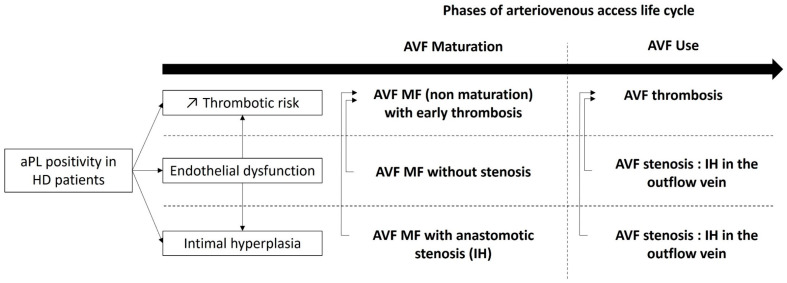
Overview of aPL putative pathophysiology in native arteriovenous fistula early and long-term complications. aPL: antiphospholipid antibody, AVF: arteriovenous fistula, HD: hemodialysis, IH intimal hyperplasia, MF maturation failure.

To our knowledge, no study has investigated the association between APS or aPL positivity and native AVF maturation failure. A cross-sectional study by Sunnesh Reddy Anapalli et al., published in 2022, found a statistically significant association between IgG and IgM and aCL and AVF failure, defined as an AVF that never went to the point of successful cannulation, or failed within the first three months. This study focusing on 50 patients with native AVF failure and 50 controls, IgG aCL and IgM aCL were associated with AVF thrombosis. AVF Maturation was not directly assessed in this study. Also, they did not mention if aCL were persistently positive and their cut off for IgG aCL and IgM aCL were > 10 GPL and > 15 MPL, respectively [45]. One clinical case reported AVF maturation failure in a patient with primary APS [58]. Our retrospective study on 103 HD patients showed a statistically significant association between AVF maturation failure (defined by the absence or a delay of maturation according to KDOQI guidelines) and aPL or APS. This association was independent of stenosis and intimal hyperplasia in a multivariate analysis. Interestingly, we reported that patients with a fluctuation aPL profiles also have a significant higher prevalence of AVF maturation failure [48]. We hypothesized that aPL might cause AVF maturation failure, possibly through endothelial dysfunction leading to an impaired vascular remodeling capability without stenosis [7,59,60]. Moreover, because APS is associated with endothelial dysfunction and intimal hyperplasia in some aPL-related manifestations [7,8,61], AVF maturation failure could be related to stenosis and intimal hyperplasia in the setting of aPL positivity. Also, AVF maturation failure could be related to early thrombosis in the setting of aPL positivity. Figure 1 summarizes the putative pathogenesis of aPL-related AVF maturation failure. Further research is needed to better understand the underlying mechanisms and to develop effective strategies for preventing and treating AVF maturation failure in patients with APS or aPL.

### 4.2. AVF Thrombosis

ESKD is associated with more thrombotic manifestations compared to the general population [62,63]. This higher risk is not fully explained by risk factors encountered in HD population. A hypercoagulability state is usually related to uremic toxins accumulation and is mediated by platelet dysfunction as well as an increased level of the procoagulant tissue factor both in vitro and in vivo [64]. Modification of the functional properties of human venous endothelial cells (VEC) and arterial endothelial cells (AEC) have been observed. Indeed, a recent study reported that VEC acquire a prothrombotic phenotype in contact with uremic serum whereas AEC acquire an inflammatory phenotype [65]. Furthermore, HD is associated with elevated levels of procoagulant factors such as prothrombin fragments and thrombin–antithrombin complexes [45]. Native AVF thrombosis remains a major cause of morbidity in HD patients, representing the most common cause of vascular access failure [66]. Such thrombotic event can occur early after AVF creation, usually due to an inflow problem (e.g., juxta anastomotic stenosis, technical errors of construction, and anatomic abnormalities) or later due to outflow vein stenosis or intimal hyperplasia [45].

The role of hereditary and acquired thrombophilia in AVF failure and AVF patency has been reported in few studies, and data suggest that most of the AVF thrombosis in the setting of thrombophilia occurs during the first month after AVF creation [40,44,45]. Because of the higher risk of arterial and venous thrombosis in patients with persistent aPL, AVF thrombosis would be expected to occur more predominantly in these patients. Indeed, several studies have described the association between aPL and AVF thrombosis whereas others did not find such association. Table 3 summarizes the available studies on aPL and native AVF thrombosis. A systematic review and meta-analysis confirmed the association between aPL (lupus anticoagulant and IgG aCL) and AVF thrombosis [15]. Non-criteria antibodies that are not included in the 2023 ACR/EULAR classification criteria for APS have been associated with AVF thrombosis such as IgA aβ2GPI [21,22,42]. On the contrary, IgG and IgM aβ2GPI have not been associated with native AVF thrombosis in the literature.

Whether AVF thrombosis in the setting of aPL positivity is favored by stenosis or intimal hyperplasia is not known. Indeed, in the setting of aPL positivity, early thrombosis could be favored by anastomotic intimal hyperplasia [67,68]. Intimal hyperplasia is a well-known non-thrombotic histological lesion of APS, well described in aPL-associated disorders such as aPL-associated nephropathy [61,69]. The latter involves the activation of the mTOR signaling pathway [10]. On the other hand, late AVF stenosis and intimal hyperplasia are caused by tissue remodeling and proliferation which may gradually progress during fistula aging and HD procedure itself (needle injury, change in blood flow, etc.). Indeed, HD duration has been described as a risk factor for AVF thrombosis [37,42].

### 4.3. Stenosis and Intimal Hyperplasia

One of the most common complications associated with a native AVF is the stenosis of the outflow vein, resulting in thrombosis, the most common cause of late AVF loss [70]. Both stenosis and thrombosis compromise AVF primary patency and usually coincide, but they cannot always be distinguished [22]. Stenosis is commonly reported in up to 60% of functional AVF. This finding is of interest because AVF thrombosis is usually attributed to the presence of venous stenosis or inflow abnormalities (anastomotic stenosis). The implementation of a surveillance program dedicated to the detection of progressive subclinical stenosis is of importance as data suggest that multiple factors are required for AVF failure [50].

A common cause of stenosis is intimal hyperplasia, which is well described in AVF [67,68]. Intimal hyperplasia is a crucial histopathological injury and forms the basis for vascular stenosis. It implies shear stress, endothelial dysfunction, inflammation, proliferation, and migration of vascular smooth muscle cells and extracellular matrix amalgamation and degradation [54]. It can take place either at anastomotic levels in the outflow vein or playing a role in restenosis after angioplasty. The prevalence of the latter complication has dropped since the use of drug coated balloon or stent mostly using paclitaxel for its anti-proliferative effect [54]. Despite the description of intimal hyperplasia in outflow veins before AVF surgical creation, studies failed to find any association between pre-existing intimal hyperplasia and AVF stenosis [53,67].

There are few studies evaluating AVF stenosis in the setting of aPL positivity in HD patients. In a combined retrospective and prospective cohort study of a single outpatient dialysis unit, the presence of IgM aCL was associated with AVF stenosis. In multivariate analysis, the presence of stenosis was significantly associated with the development of AVF thrombosis [71]. To our knowledge, intrastent restenosis or restenosis after drug-eluted balloon have not been studied in aPL positive HD patients. However, few studies have demonstrated that patients with APS are predisposed to high rates of restenosis of the coronary arteries after percutaneous coronary intervention [72]. As previously said, intimal hyperplasia is a well-known non-thrombotic histological lesion associated with aPL positivity involving the activation of the mTOR signaling pathway [10]. Up to now, no studies comparing AVF restenosis and drug-eluted angioplasty (e.g., sirolimus coated balloon angioplasty) in aPL positive HD patients are currently available.

### 4.4. Mortality

Serrano et al. reported in a 2-year prospective study that IgA aβ2GPI positivity was associated with a higher mortality rate, compared to negative patients [21]. However, other authors found no association with mortality [16].

## 5. Antiphospholipid Antibody Testing before AVF Creation

Routine thrombophilia screening is not recommended before HD vascular access surgery [22,73]. However, certain risk factors, such as history of prior AVF failure, history of unprovoked venous or arterial thrombosis, especially at a young age, should raise the suspicion of thrombophilia. Because aPL positivity appears as an additional risk indicator for AVF failures, we would recommend performing aPL screening, when possible, as a preoperative test. Indeed, most of the time, first laboratory assessment for thrombophilia occurs after the occurrence of an AVF complication, making the interpretation challenging. Knowing aPL status could also help clinicians to select the optimal perioperative therapy in order not only to promote AVF maturation, but also to guide the surveillance of the vascular access.

## 6. Treatment Options

Despite the high prevalence of aPL in HD populations, there are no specific treatment guidelines for aPL associated AVF complications. Treatment options should follow HD guidelines for medical or surgical treatments and endovascular interventions promoting AVF maturation and surgical and endovascular interventions for non-maturing AVF and for AVF surveillance with the objective to maintain long-term AVF patency [73].

Because of accelerated atherosclerosis in patients with persistent aPL, cardiovascular risk factors should be considered for the prevention or treatment of AVF complications. In aPL-positive patients without a history of thrombosis, low-dose aspirin should be balanced with bleeding risks for primary prevention of thrombotic manifestation, although its effect is uncertain on AVF maturation [73].

Regardless of other thrombotic manifestations, whether vitamin K antagonists (VKA) should be initiated after AVF thrombosis in the setting of aPL positivity is not known. Major bleeding complications have been associated with the use of VKA in the HD population [74]; VKA also have detrimental effects on AVF remodeling process by promoting intimal hyperplasia and calcification [75]. Despite the superior benefit-risk profile of direct oral anticoagulants versus VKA observed in the HD population, the use of the latter anticoagulant should be avoided because of the increased risk of arterial thrombosis in APS patients [76]. Low-molecular-weight-heparin could be a reasonable option in these patients. Further studies are needed to answer these questions.

Potential future therapeutic strategies should focus on targeting endothelial dysfunction, intimal hyperplasia, or the coagulation system depending on the vascular access complication. Also targeting mTOR pathway might be a therapeutic option in selected patients. However, more studies are needed because the lack of knowledge in the pathophysiology of aPL associated AVF thrombosis, stenosis, or maturation failure.

## 7. Conclusions

Antiphospholipid antibody positivity is frequent in HD patients, and its prevalence is higher than in the general population or in the conservatively treated ESKD. Persistent positivity of aPL is associated with native AVF thrombosis, and aCL and LA positivity seem to be particularly associated with AVF thrombosis. However, data are lacking regarding AVF maturation and stenosis. Further studies are necessary in order to clarify the association between aPL and stenosis or maturation failure, but also to develop effective strategies for preventing and treating AVF maturation failure in patients with APS or aPL.

## Figures and Tables

**Table 1:** Main differences between 2006 revised Sapporo and 2023 ACR/EULAR classification criteria for antiphospholipid syndrome.

	2006 Revised Sapporo	2023 ACR/EULAR
**Classification**	At least 1 clinical criterion AND 1 laboratory criterion	3 points from clinical domains AND at least 3 points from laboratory domains
**Clinical criteria**		Entry criteria and scoring: count the highest weighted criterion towards the total score
	2 clinical criteria 1. Vascular thrombosis: One or more clinical episodes of arterial, venous, or small vessel thrombosis, in any tissue or organ 2. Pregnancy morbidity	6 clinical domains 1. Macrovascular-Venous Thromboembolism 2. Macrovascular-Arterial Thrombosis 3. Microvascular 4. Obstetric 5. Cardiac Valve 6. Hematology
Considered as non-criteria-manifestations:		
- Heart valve disease	Yes	No
- Livedo racemosa	Yes	No
- Thrombocytopenia	Yes	No
- Nephropathy,	Yes	No
- Neurological manifestations	Yes	Yes
- Pulmonary/Adrenal hemorrhage	Yes	No
**Laboratory criteria**		
Persistent positivity (at 12 weeks)	Yes	Yes
Timeline of aPL positivity and clinical criteria	Less than 5 years of clinical criteria	Within 3 years of clinical criterion
Thresholds of aCL and/or aβ2GPI	aCL: >40 GPL or MPL, or >the 99th percentile aβ2GPI: >the 99th percentile	aCL or aβ2GPI: Moderate 40–79 units High >80 units
Antibodies for laboratory criteria:		
- Positive LA	Yes	Yes
- IgG aCL or aβ2GPI	Yes	Yes
- IgM aCL and/or aβ2GPI	Yes	Yes. If isolated: are not sufficient (weight only 1 point)

aCL: anticardiolipin antibody, aβ2GPI: anti-β2 glycoprotein I antibody, LA: lupus anticoagulant.

**Table 2:** Summary of the literature on aPL-associated AVF thrombosis.

Year	First Author (Reference)	Study Type, *n*	Follow Up (Months)	aPL	Cut-Off	aPL Confirmation	Proportion of Native AVF	AVF Thrombosis and Outcomes
1991	F. Garcia-Martin [30]	Retrospective *n* = 51	NA	LA, IgG aCL	12.5 GPL U/mL	NA	NA	IgG aCL: Higher incidence of early (6 to 96 h) thrombosis in IgG aCL (31%) versus control (17%). Concentrations of IgG aCL early AVF thrombosis were significantly greater than in patients without it (18.5 ± 7.4 GPL U/mL versus 7.4 ± 0.8, *p* < 0.001).
1992	S. L. Chew [16]	Prospective *n* = 60	12	LA, IgG aCL	10 GPL	Yes	100%	LA, IgG aCL: no association with AVF thrombosis or death during the 12 months follow up.
1995	P. Brunet [19]	Cross-sectional *n* = 97	6	LA, IgG aCL	20 GPL	NA	81.40%	LA: association with AVF thrombosis, IgG aCL: no association with AVF thrombosis
1995	R. Prakash [20]	Retrospective *n* = 17	30	IgG aCL	23 GPL	NA	100%	No events of AVF thrombosis were encountered during the period of review.
1999	J. George [34]	case–control *n* = 81	NA	aCL, aβ2GPI	NA	NA	6.2%	aCL, aβ2-GPI: no association with AVF thrombosis
1999	B. J. Manns [35]	Cross-sectional *n* = 118	36	IgG aCL	low, moderate, and highly positive as follows: 11 to 20 GPL, 21 to 80 GPL, and more than 80 GPL	NA	75%	IgG aCL: not associated with AVF thrombosis
2000	Y.S. Haviv [31]	Retrospective *n* = 54	NA	IgG and IgM aCL, IgG and IgM aβ2-GPI	10 IU/mL	NA	31.50%	IgG and IgM aCL: association with AVF occlusion (thrombosis or IH). IgG and IgM aβ2-GPI: not associated with occlusion
2002	I. Palomo [36]	Retrospective *n* = 208	NA	aCL, aβ2-GPI and aPS	3 SD above the average of the normal controls	NA	100%	aPL: no association with AVF thrombosis.
2003	M.R.N. Nampoory [37]	Retrospective *n* = 82	NA	LA, IgG and IgM aCL, IgG and IgM aPS	IgG aCL: ≥23 GPU, IgM aCL: ≥11 MPU, IgG aPS: ≥17 GPS, IgM aPS: ≥23 MPS	NA	70.70%	LA: association with AVF thrombosis, IgG, IgM aCL and IgG, IgM aPS: no association with AVF thrombosis
2003	Y-C. Chuang [38]	Cross-sectional *n* = 48	NA	IgG aCL	12 GPL-Uuml	NA	52.10%	No IgG aCL positivity in this cohort
2004	D.Molino [39]	Retrospective *n* = 40	NA	LA, IgG and IgM aCL, Ig G and IgM aPT	NA	NA	100.00%	Ig G, IgM aPT, IgG, IgM aCL: significantly associated with AVF thrombosis
2005	F-R. Chuang [27]	Cross-sectional *n* = 483	NA	IgM aCL	IgM aCL: 6 GPL-U/mL	NA	72.30%	IgM aCL: not associated with AVF thrombosis
2005	G. A. Knoll [40]	Case-control *n* = 419	NA	LA, IgG aCL, IgM aCL	IgG, IgM aCL: medium titer of 30 GPL or MPL U/mL	No	91.40%	aCL: not associated with AVF thrombosis
2005	F. Gültekin [41]	Retrospective *n* = 103	NA	IgG, IgM aCL	NA	NA	100%	IgG, IgM aCL: not associated with AVF thrombosis
2006	J. Roozbeh [17]	Prospective *n* = 171	14	IgG aCL	Negative: <10 GPL. Low positive: 10 ≤ aCL < 20 GPL, Medium positive: 20 ≤ aCL < 40 GPL, and highly positive: ≥ 40 GPL units.	NA	100%	IgG aCL: not associated with AVF thrombosis
2009	S. Ozmen [18]	Cross-sectional *n* = 103	NA	IgG, IgM aCL	NA	NA	NA	Not associated with AVF thrombosis, and AVF survival
2012	A. Serrano [21]	Prospective *n* = 124	24	IgG, IgM, IgA aCL, IgG, IgM, IgA aβ2-GPI	20 U/mL	Yes	100%	IgA aβ2-GPI: associated with AVF thrombosis, cardiovascular disease and mortality
2013	B. Salmela [22]	Retrospective *n* = 219	NA	LA, IgG aCL, IgG aβ2-GPI	15 U/mL	NA	100%	aPL: not associated with AVF failure (thrombosis or stenosis)
2013	S. Hadhri [42]	Case-control *n* = 101	NA	LA, IgG, IgM, IgA aCL, IgG, IgM, IgA aβ2-GPI	95th percentile for healthy blood donors (7 MPL/mL, 10 GPL/mL and 10 APL/mL for IgM, IgG and IgA aCL, respectively, and 8 U/mL for IgM, IgG and IgA anti-β2-GPI)	NA	100%	IgA aβ2-GPI: independent risk factors for AVF thrombosis (OR = 3.4; 95% CI, 1.21 to 9.55; *p* = 0.02)
2014	S. Bataille [28]	Retrospective *n* = 192	NA	LA, IgG and IgM aCL, IgG and IgM aβ2-GPI	aCL and aβ2-GPI: 99e percentile	NA	68%	aPL and LA: significantly associated with AVF thrombosis
2016	F. I. Fadel [43]	Prospective *n* = 50	48	IgG aCL	NA	NA	80%	IgG aCL: significantly associated with AVF thrombosis.
2019	C. Grupp [44]	Prospective *n* = 70	384	LA, IgG, IgM, and IgA aCL	Respectively 12 GPLU/mL, 6 MPLU/mL, and 10 APL U/mL	No	100%	LA, IgG, IgA, IgM aCL: significantly associated with AVF thrombosis. Patient survival tended to be shorter in patients aPL than in control group, but without statistical significance
2022	S. R. Anapalli [45]	Cross-sectional *n* = 100	NA	IgG and IgM aCL	Respectively 10 and 15 MPL units	NA	100%	IgG and IgM aCL: significantly associated with AVF thrombosis (*p* value < 0.001)
2020	P.R J. Ames [15]	systematic review and meta-analysis IgG aCL: n = 1554, LA: *n* = 511	NA	LA, IgG aCL	NA	NA	NA	IgG aCL and LA associated with AVF thrombosis

aCL: anticardiolipin antibodies, aPS: antiphosphatidyl serin antibody, aPT: antiprothrombin antibodies, AVF: arteriovenous fistula, aβ2-GPI: anti- β2 Glycoprotein I antibodies, LA: lupus anticoagulant, NA: not available.

**Table 3:** Summary of the articles evaluating the association between the criteria and non-criteria aPL and AVF thrombosis. The cross symbol designates the absence of association between aPL and AVF thrombosis, whereas the check symbol designates an association. Question marks represent studies in which association between aPL and AVF thrombosis in non-interpretable because of the absence of thrombosis or the absence of aPL positivity.

				Association with AVF Thrombosis					
Year	First Author (References)	Study Type	*n*	LA	IgG aCL	IgM aCL	IgG aβ2GPI	IgM aβ2GPI	Other
1991	F. Garcia-Martin [30]	retrospective	51	x	✓				
1992	S. L. Chew [16]	prospective	60	x	x				
1995	P. Brunet [19]	cross-sectional	97	✓	x				
1995	R. Prakash [20]	retrospective	17		?				
1999	J. George [34]	case-control	81		x		x		
1999	B. J. Manns [35]	cross-sectional	118		x				
2002	Y.S. Haviv [31]	retrospective	54		✓	✓	x	x	
2002	I. Palomo [36]	retrospective	208		x		x		aPS
2003	M. R.N. Nampoory [37]	retrospective	82	✓	x	x			aPS
2003	Y-C. Chuang [38]	cross-sectional	48		?	?			
2004	D. Molino [39]	retrospective	40	x	✓	✓			
2005	F-R. Chuang [27]	cross-sectional	483			x			
2005	G. A. Knoll [40]	case-control	419	x	x	x			
2005	F. Gültekin [41]	retrospective	103		x	x			
2006	J. Roozbeh [17]	prospective	171		x				
2009	S. Ozmen [18]	cross-sectional	103		x	x			
2012	A. Serrano [21]	prospective	124		x	x	x	x	IgA aβ2GPI
2013	B. Salmela [22]	retrospective	219	x			x	x	
2013	S. Hadhri [42]	case-control	101						IgA aβ2GPI
2014	S. Bataille [28]	retrospective	192	✓					
2016	F. I. Fadel [43]	Prospective	55		✓				
2019	C. Grupp [44]	prospective	70	✓	✓	✓			IgA aCL
2022	S. R. Anapalli [45]	cross-sectional	100		✓	✓			
2020	P. R. J. Ames [15]	meta-analysis	1554	✓					
			511		✓				

aCL: anticardiolipin antibody, aPS: antiphosphatidyl serin antibody, AVF: arteriovenous fistula, aβ2GPI: anti-β2 glycoprotein I antibody, LA: lupus anticoagulant.

## Data Availability

No new data were created or analyzed in this study. Data sharing is not applicable to this article.
